# Population Density Modulates Drug Inhibition and Gives Rise to Potential Bistability of Treatment Outcomes for Bacterial Infections

**DOI:** 10.1371/journal.pcbi.1005098

**Published:** 2016-10-20

**Authors:** Jason Karslake, Jeff Maltas, Peter Brumm, Kevin B. Wood

**Affiliations:** 1 Department of Biophysics, University of Michigan, Ann Arbor, MI; 2 Department of Physics, University of Michigan, Ann Arbor, MI; MIT, UNITED STATES

## Abstract

The inoculum effect (IE) is an increase in the minimum inhibitory concentration (MIC) of an antibiotic as a function of the initial size of a microbial population. The IE has been observed in a wide range of bacteria, implying that antibiotic efficacy may depend on population density. Such density dependence could have dramatic effects on bacterial population dynamics and potential treatment strategies, but explicit measures of per capita growth as a function of density are generally not available. Instead, the IE measures MIC as a function of initial population size, and population density changes by many orders of magnitude on the timescale of the experiment. Therefore, the functional relationship between population density and antibiotic inhibition is generally not known, leaving many questions about the impact of the IE on different treatment strategies unanswered. To address these questions, here we directly measured real-time per capita growth of *Enterococcus faecalis* populations exposed to antibiotic at fixed population densities using multiplexed computer-automated culture devices. We show that density-dependent growth inhibition is pervasive for commonly used antibiotics, with some drugs showing increased inhibition and others decreased inhibition at high densities. For several drugs, the density dependence is mediated by changes in extracellular pH, a community-level phenomenon not previously linked with the IE. Using a simple mathematical model, we demonstrate how this density dependence can modulate population dynamics in constant drug environments. Then, we illustrate how time-dependent dosing strategies can mitigate the negative effects of density-dependence. Finally, we show that these density effects lead to bistable treatment outcomes for a wide range of antibiotic concentrations in a pharmacological model of antibiotic treatment. As a result, infections exceeding a critical density often survive otherwise effective treatments.

## Author Summary

The pace of antibiotic discovery has rapidly slowed in the last few decades, creating an urgent need to reevaluate and optimize therapies based on current drugs. In this work, we combine quantitative laboratory experiments on bacterial populations with mathematical models of antimicrobial therapies to demonstrate that bacterial populations of different sizes (densities) may respond very differently to the same antibiotic treatment. As a result, otherwise successful antibiotic treatments may fail to eradicate bacterial infections that have reached a critical population density. Our findings indicate that it is important to consider the size of a bacterial infection when designing effective antimicrobial therapies.

## Introduction

The inhibitory effects of antibiotics often decrease with increasing density of the starting microbial population, a phenomenon known as the inoculum effect [1]. While the IE is commonly attributed to enzymatic degradation of the drug [2, 3]—with the classical example being the degradation of β-lactams by a β-lactamase enzyme—recent studies have pointed to a range of other potential mechanisms, including heat-shock mediated growth bistability [4], intercellular signaling between resistant and sensitive cells [5], and a decrease in per-cell antibiotic concentration and therefore the number of available drug molecules per cell [6]. However, the consequences of the inoculum effect for microbial population dynamics, the design of optimal treatment strategies, and the evolution of resistance are debated, in part because antibiotic efficacy is typically measured as a function of *initial* population (inoculum) size, not as an explicit function of population density. Specifically, the IE is commonly described as an increase in the minimum inhibitory concentration (MIC) of a drug as a function of inoculum size [1]. The MIC measurement requires cell populations to grow above a particular threshold level—measured by optical density or colony growth on solid media—meaning that cell density is changing by several orders of magnitude on the timescale of the experiment. In addition, the outcome measure in such experiments is binary, with cell populations either surviving or dying. Because clinical therapies often involve time-dependent drug doses that span both sub- and super-MIC levels [7], it is difficult to systematically evaluate and optimize these dosing regimens without knowing the direct functional relationship between per capita growth rate, cell density, and drug concentration.

Despite these inherent challenges, several recent studies have shown that a deeper quantitative understanding of the IE holds the promise of improved strategies for minimizing microbial growth or mitigating the evolution of resistance. For example, certain classes of protein synthesis inhibitors were recently shown to induce growth bistability, leading to a strong IE and population growth that depends markedly on the frequency of periodic drug dosing [4]. In cases where the IE can be traced to enzyme production, mathematical models suggest that optimized treatment protocols may resurrect otherwise ineffective first line drugs and prolong the efficacy of newer antibiotics [8]. In addition, intuitive treatment strategies—such as introducing an inhibitor of the enzyme—may actually promote, rather than inhibit, the spread of resistance [3], while sophisticated measures of drug efficacy, such as the MIC of a single cell, may be required for predicting selection pressures driving resistance [9]. Even in the absence of detailed mechanistic information, pharmacological models based on classical MIC measurements suggest that otherwise successful therapies may fail to clear some infections [6]. Taken together, these studies suggest that the IE may play an important role in optimizing treatment strategies. Unfortunately, despite the promise of these emerging approaches, our quantitative understanding of the IE remains limited because the functional relationship between population density and drug inhibition is generally not known.

To address these issues, we constructed multiplexed, computer-automated culture devices that allow us to monitor per capita growth in real time while maintaining bacteria at fixed population densities. The use of continuous culture devices in microbiology dates back more than 60 years [10], and constant density bioreactors (“turbidostats”) have proven useful in a wide range of microbiology settings [11]. Unfortunately, commercially available bioreactors are expensive and somewhat inflexible, making the use of bioreactors far less common than traditional batch culture methods. Recently, however, a number of simple but elegant reactor designs has reinvigorated interest in continuous culture devices for applications ranging from synthetic circuit characterization [12] to cancer biology [13], stochastic gene expression [14], and antibiotic resistance under sustained selection pressure [15, 16]. Inspired by these studies, we developed a simple, multiplexed turbidostat that would allow us to measure the growth of bacterial populations at fixed densities in response to a constant concentration of antibiotics. Our goal is to strip away technical ambiguities associated with classical measurements of the IE and provide the functional relationship between antibiotic inhibition and population density. In turn, we incorporate this density dependence into mathematical models to demonstrate its marked effects on population dynamics and potential treatments.

While the IE has been observed across a wide range of bacterial species, here we focus on *E*. *faecalis*, a gram-positive species increasingly recognized as a clinically important pathogen [17]. *E*. *faecalis* underlies a host of nosocomial infections, and they rapidly acquire high-level resistance through genome mutations and horizontal gene transfer, making them important contributors to the spread of drug resistance in other bacteria [17–21]. In addition, *E*. *faecalis* are known to participate in community-like behavior, including cell-cell communication via two-component signaling [22–24], sharing of extracellular DNA via fratricide [25, 26], phage-mediated gene transfer [27], pheromone-induced quorum sensing [28, 29], and chromosomal transfer [30]. These studies offer molecular evidence of widespread intercellular interactions in *E*. *faecalis* populations, leading us to hypothesize that the behavior of these communities—in particular, their responses to antibiotic treatments—may depend strongly on population density. Indeed, the classical IE has been observed in *E*. *faecalis* for a wide range of antibiotics [31–33], but little is known about how such density dependence might impact optimal therapies or ecological and evolutionary dynamics.

## Results

### Continuous culture devices allow for explicit measurements of density-dependent cell growth

**Fig 1:**
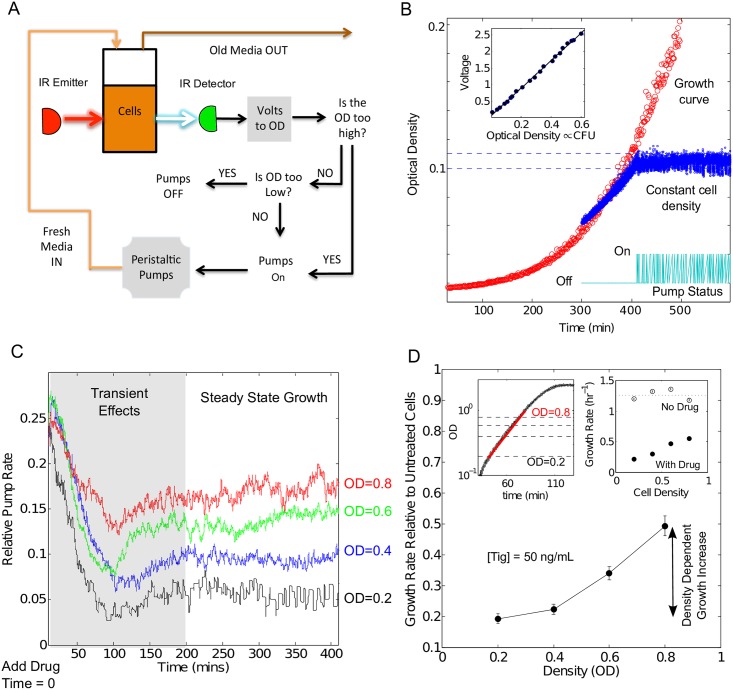
Computer-controlled continuous culture devices can measure population growth at constant cell density. A. Bacterial cultures (15 mL) are grown in glass vials with customized Teflon tops that allow inflow and outflow of fluid via silicone tubing. Cell density is monitored by light scattering using infrared LED/Detector pairs on the side of each vial holder. At the onset of the experiment, stationary phase bacterial cultures are diluted 500X and allowed to grow in the culture vials until a specific density is reached. At that point, drug is manually added at the desired concentration to both the culture vial and a connected chamber with fresh media. Flow between the media chamber, the culture vial, and a waste vial is managed by a series of computer-controlled peristaltic pumps that maintain constant cell density according to the pictured schematic. The entire system is controlled by custom Matlab software, and up to 18 cultures can be grown simultaneously using a multi-position magnetic stirrer. See also Figure A in S1 Text. B. Examples of a bacterial growth curve (red) and a constant cell-density experiment in which feedback from light scattering is used to maintain a constant cell density (blue). Lower inset: time series showing the status of the inflow/outflow pump, which provides fresh media and removed waste, during constant density (blue) experiment. Because total culture volume remains constant, the pump status time series can be used to calculate per capita growth rate, g, as a function of time when cell density is held constant: g = F/V, where F is the (time dependent) pump flow rate (mL/min) and V is the (constant) culture volume (mL). Upper inset: Calibration plot showing that voltage output from IR detectors is linearly related to optical density. C. Time dependent population growth rate is estimated from the relative pump flow rate F(t), which is the flow rate of the pumps required to maintain cell density (flow rates measured relative to maximum possible flow rate of approximately 1 ml/min). Drug is added at time 0, and following transient growth rate dynamics of approximately 200 minutes, growth rate reaches a steady state that is dependent on cell density. To reduce high-frequency noise, F(t) is estimated with a moving-average filter with window size of 15 minutes. D. To estimate growth rate relative to untreated cells, the steady state growth rate F(t) is averaged in the steady state and normalized by the same measurement in the absence of drug. Upper left inset, full growth curve for *E*. *faecalis* in the absence of drug. The densities measured here (0.2≤OD≤0.8) correspond to exponential phase growth, represented by a straight line (red) on a semi-log plot. Upper right inset, growth rate (not normalized) with and without drug. Without drug, growth varies by approximately 6% around the mean over these density ranges.

To determine explicitly the density dependence of single drug activity, we built multiplexed computer-automated microbial culture chambers (Fig 1, Figure A in S1 Text) that maintain populations of *E*. *faecalis* at fixed densities while exposing them to different concentrations of antibiotics (Methods). Cell density is monitored by light scattering and maintained through feedback control using a series of peristaltic pumps, which deliver fresh media and drugs and remove waste (Fig 1A). Population growth (specifically, per capita growth) over time can then be calculated by monitoring the flow rates of the pumps (Fig 1B–1D). By repeating this procedure at multiple cell densities—each in the exponential growth phase (Fig 1D, inset)—we can directly measure the density dependence of antibiotic inhibition for any given drug concentration (Fig 1D).

### Changes in cell density can increase or decrease the efficacy of antibiotics

**Fig 2:**
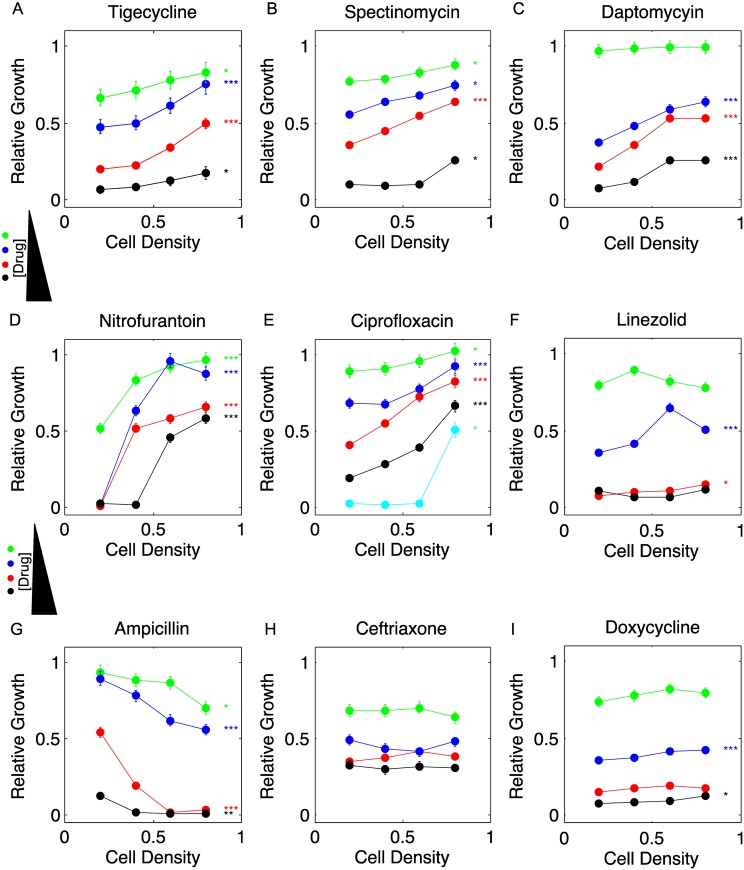
Cell density modulates the inhibitory effects of multiple antibiotics. A-I: Steady state population growth rate (relative to untreated cells) as a function of cell density for multiple drug concentrations. Drug concentrations are A. Tigecycline concentration = 15 (green), 25 (blue), 50 (red), 100 (black) ng/mL; B. Spectinomycin concentration = 50 (green), 100 (blue), 150 (red), 400 (black) μg/mL; C. Daptomycin concentration = 1.0 (green), 1.25 (blue), 1.50 (red), 3.0 (black) μg/mL; D. Nitrofurantoin concentration = 50 (green), 100 (blue), 125 (red), 250 (black) μg/mL; E. Ciprofloxacin concentration = 100 (green), 150 (blue), 200 (red), 300 (black), 400 (cyan) ng/mL; F. Linezolid concentration = 0.1 (green), 0.5 (blue), 4 (red), 5 (black) μg/mL; G. Ampicillin concentration = 200 (green), 300 (blue), 400 (red), 500 (black) ng/mL; Note that ampicillin growth does not reach steady state on the time-scale of our experiment, so these measurements are effective growth rates averaged over a non-steady state (Figure A in S1 Text). H. Ceftriaxone concentration = 5 (green), 50 (blue), 200 (red), 300 (black) μg/mL; I. Doxycycline concentration = 33 (green), 100 (blue), 333 (red), 500 (black) ng/mL. Statistically significant differences between growth at lowest and highest densities (0.2 and 0.8), intermediate densities (0.4 and 0.6), or both are indicated by *, **, and ***, respectively. Error bars are +/- 1.96 standard error (95% confidence intervals). See also Figures B, C in S1 Text.

We measured the density dependence of nine antibiotics (Table A of S1 Text), many of which represent current treatment options for *E*. *faecalis* infections [18]. Interestingly, we find that many antibiotics (e.g. tigecycline) exhibit density-dependent inhibition, while others (e.g. ceftriaxone) exhibit inhibition that is largely independent of density (Fig 2). While most drugs show decreased inhibitory activity as density increases—consistent with interpretations of the classical IE—ampicillin exhibits increased inhibition at higher densities. Of the nine drugs we tested, seven (tigecycline, spectinomycin, daptomycin, nitrofurantoin, ciprofloxacin, linezolid, and doxycycline) show a clear density dependent decrease in inhibition for at least one dosage, while ampicillin shows an increase in efficacy. In addition, one can estimate the drug’s half-maximal inhibitory concentration (K) at each density by fitting the growth, g, at each density to a sigmoidal dose-response function, $g={\left(1+{\left(D/K\right)}^{h}\right)}^{-1}$, where D is the drug concentration and h is a Hill-like steepness coefficient [34]. We find that six of the nine drugs show statistically significant change in K, and K can increase by a factor of three or more over this rather narrow density range (Figure C of S1 Text).

### Density-driven pH changes as a community mechanism to modulate drug efficacy

The observed density dependence of antibiotic inhibition may result from multiple different mechanisms, perhaps in combination [2–6]. Because *E*. *faecalis* are fermentative bacteria and pH is known to modulate antibiotic efficacy in a wide range of pathogens [31, 35], we asked whether density dependence could be partially explained by community-driven changes in local pH during exponential phase growth. While pH is known to modulate drug efficacy and to depend on bacterial growth phase, it has somewhat surprisingly not been linked with the IE, perhaps because IE measurements typically standardize only the initial pH (which, like density, may change significantly during the experiment).

**Fig 3:**
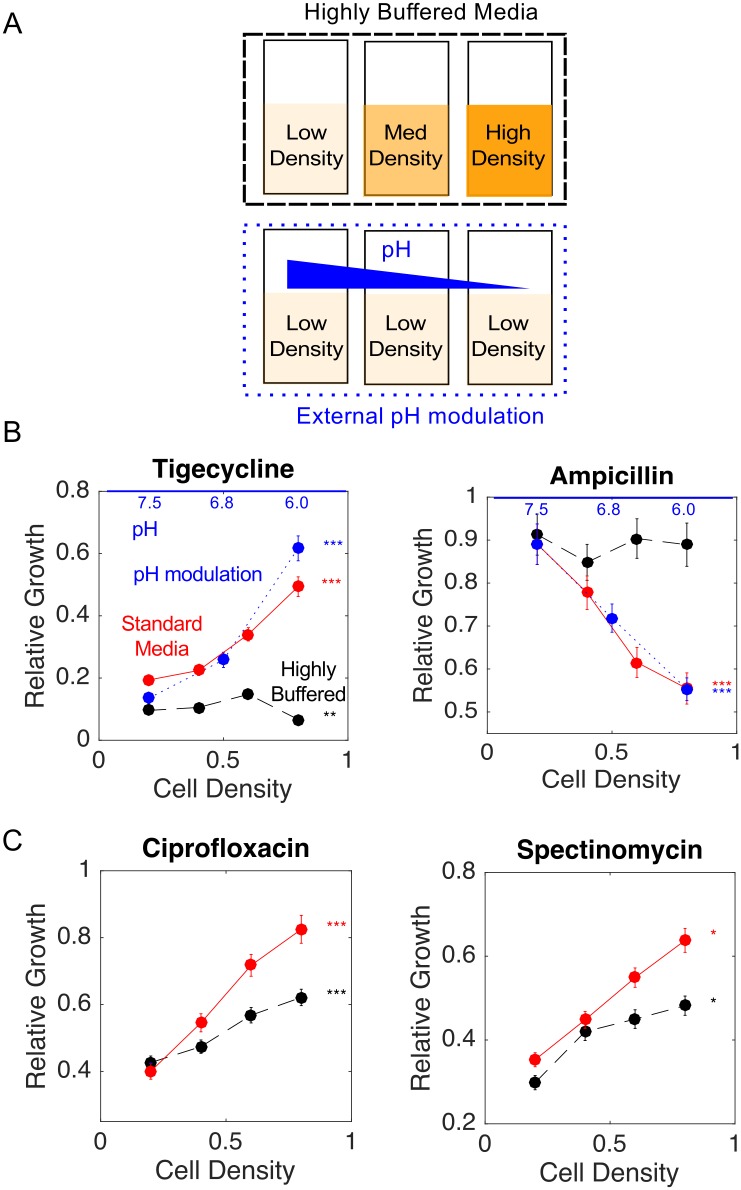
Density dependence of antibiotic inhibition partially due to local pH changes. A. Top row: Steady state population growth was measured as a function of cell density (here schematically represented by low, medium, and high density) by holding each vial at a constant density while exposing cells to constant drug concentration in highly buffered media. Bottom row: Different culture vials were all held at low-density (OD = 0.2) but grown in BHI supplemented with HCl to achieve pH = 7.5, 6.8, and 6.0, which correspond to pH of steady state cultures held at OD = 0.2, 0.5, and 0.8, respectively. B. Red curves, regular media. Black dashed curves, buffered media. Blue dotted curves, external pH modulation. Tigecycline concentration 50 ug/mL; Ampicillin concentration 200 ng/mL;C. Red curves, regular media. Black dashed curves, buffered media. Ciprofloxacin concentration 200 ng/mL, Spectinomycin concentration 150 ug/mL. Statistically significant differences between growth at lowest and highest densities (0.2 and 0.8), intermediate densities (0.4 and 0.6), or both are indicated by *, **, and ***, respectively. See also Figure D in S1 Text. Error bars are +/- 1.96 standard error (95% confidence intervals).

Indeed, we found that exchanging regular media for highly buffered media (see Methods) modified the density dependence for some but not all drugs. For example, the density dependence was almost completely eliminated in highly buffered media for tigecycline and ampicillin (Fig 3B). Note that growth in buffered and regular media did not differ significantly in the absence of drug (Figure D in S1 Text, left panel). Furthermore, external modulation of pH recapitulated the effect in low density cultures (Fig 3B). On the other hand, ciprofloxacin and spectinomyin continued to show significant, but decreased, density dependence in highly buffered media (Fig 3C). Spectinomycin belongs to a class of protein synthesis inhibitors recently shown to exhibit an IE due to heat-shock mediated growth bistability [4], and indeed spectinomcyin inhibition remained strongly density-dependent even in buffer, though the dependence was slightly weaker at high densities. At the other extreme, highly buffered media actually led to increased density dependence for ceftriaxone, indicating that changes in pH may counteract a second, unknown mechanism that decreases drug efficacy at high densities (Figure D in S1 Text, right panel). Our results are also consistent with recent measurements in uropathogenic strains that show decreasing external pH will increase MIC for ciprofloxacin while decreasing MIC for ceftriaxone and ampicillin in disc diffusion assays [35]. These measurements indicate that density-mediated changes in pH represent one mechanism for the IE during exponential phase growth. For some drugs (ampicillin and tigecycline), these pH changes account for the majority of the observed density-dependent changes, while for other drugs (ciprofloxacin, spectinomycin, ceftriaxone), additional mechanisms appear to dominate.

### Density-dependent drug efficacy impacts growth dynamics in constant drug environments

Because our experiments provide explicit measurements of per capita growth as a function of population density, we can quantify the impact of cell density on specific growth dynamics or treatment strategies. For example, what effect does the observed density dependence have on the time it takes a growing population to reach a particular threshold size in a constant drug environment? To answer this question, we first developed a simple mathematical model for density-dependent turbidostat growth. Our model incorporates density-dependent inhibition as an increase or decrease in the effective drug concentration, a process governed by a phenomenological rate constant ε (Methods; S1 Text). This change in drug concentration could represent, for example, the biochemical degradation of drug by enzymes, though recent work has shown that concentration rescaling can also describe changes in drug efficacy from a wide range of other sources, including genetic mutations and drug interactions [36, 37]. We can directly estimate ε from the steady-state turbidostat data for each drug (Table B of S1 Text); ε quantifies the rate of change in the effective drug concentration—and therefore drug efficacy—due to cell density.

Specifically, we assume that the effective drug concentration decays according to dD/dt = –εDn^j^, where ε is the phenomenological rate constant, n is the cell density, and j is a positive integer that describes the kinetic order of the decay (j = 1 is linear with cell density, j = 2 quadratic; see S1 Text). We find both models (j = 1 or j = 2) provide qualitatively accurate descriptions of density dependent turbidostat data for the drugs in this study (Figure E of S1 Text). For 5 of the 9 drugs, the quadratic model (j = 2) is quantitatively superior to the linear model (j = 1) according to standard model selection methods (S1 Text, including Table B), so we adopt that model in what follows (though we note that both models provide qualitatively similar behavior; see Figure G in S1 Text). We stress that all information about density dependence is contained in the rate constant ε (S1 Text, including Table B and Figures E, F). For example, the density dependence of tigecycline is well-described by a quadratic (j = 2) decay with ε = 0.9±0.1 (the units of ε are ([time][cell density]^2^) ^-1^, where time is measured in units of inverse per capita growth rate without drug, and cell density is measured in units of OD). The model provides a good qualitative and quantitative fit to our growth rate measurements (Figures E and F of S1 Text).

To explore growth in a constant drug environment, we next incorporated these density effects into a classic logistic model of population growth [38] that includes a Hill-like dose-inhibition curve [34] (Methods). We estimated parameters for logistic growth from drug-free growth curves and parameters of the (low density) dose-response function from a series of standard growth curves in early exponential phase (OD<0.2) at different drug concentrations. For example, for tigecycline in regular media, the dose-response curve is described by half-maximal inhibitory concentration K_0_ = 19.3±1 ng/mL and Hill coefficient h = 2.1±0.1. (Figure F of S1 Text; estimates include ± standard error of fitting parameter; see also Methods). As in the turbidostat model, we incorporated changes in drug efficacy due to population density, n, by assuming that effective drug concentration changes according to the measured rate parameter ε.

**Fig 4:**
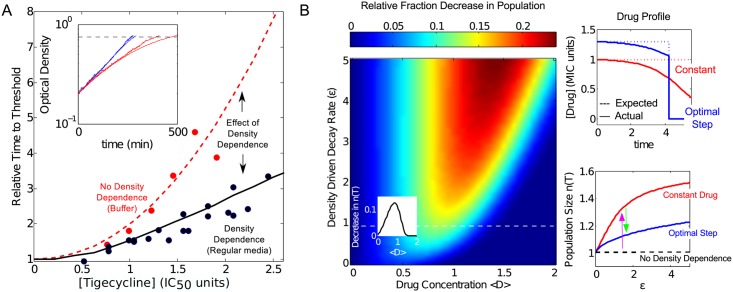
Density dependent growth modulates time-to-threshold and optimal antibiotic treatment for in-vitro growth model. **A.** Time for population to grow from OD = 0.2 to OD = 0.8 (“threshold”) in the presence of tigecycline, relative to time with no drug. Curves represent theoretical predictions with (ε = 0.9, black, solid) and without (ε = 0, red, dashed) density dependence. Points represent experimental measurements in regular media (black) and highly buffered media (red). Inset: comparison of theory (smooth lines) and experimental time series of optical density in regular media (blue) and buffer (red) for [tigecycline] = 1.2 (units of IC_50_). **B.** Decrease in final population size when naïve dosing (upper right inset, dashed red line) at initial concentration D_0_ is replaced by optimal step-like dosing (upper right inset, dashed blue line). Dashed white line: ε = 0.9, as for tigecycline. Small inset: Fraction decrease in the population as a function of <D> for tigecycline (ε = 0.9). The step-like therapy introduces drug at initial concentration D_0_/τ and then sets drug concentration to zero at time t = τT. The parameter τ is chosen to minimize the cell density n(T) at time T, the end of the treatment (0≤τ≤1). In the absence of density dependence, both therapies result in a time-averaged drug concentration $\left(\langle D\rangle =\frac{1}{T}\int_{0}^{T}D(t)dt=D_{0}\right)$. Upper right inset: drug concentration over time with (“actual”; solid lines) and without (“expected”; dashed lines) density dependence for naïve (red) and step-like (blue) dosing. Lower right inset: final population size (relative to the case with no density dependence) for the naïve treatment (red) and the optimal step-like treatment (blue) as a function of ε. At a given value of ε, density-dependence can significantly increase n(T) (magenta arrow), but the optimal step dosing can often reduce the effects by 50% or more (green arrow).

Using this simple mathematical model, we calculated the time required for a population with initial density of OD = 0.2 to reach a threshold of OD = 0.8 for different concentrations of tigecycline, which shows marked density-dependence (ε = 0.9 ±0.1; see Fig 2 and Table B of S1 Text). Our model predicts that the time to threshold increases by a factor of approximately three as tigecycline concentration is increased from 0 to 2.5 K_0_, and we were able to verify these predictions experimentally (Fig 4A). To estimate the impact of density-dependence on time to threshold, we then repeated this calculation with ε = 0 (no density dependence) but all other parameters unchanged. Even over this somewhat limited density range (0.2 ≤ OD ≤ 0.8), the density-dependence of drug efficacy can lead to a significant (approximately two-fold) decrease in time to threshold over a range of drug concentrations. Furthermore, because the density dependence of tigecycline is largely eliminated in highly buffered media (Fig 3), we experimentally confirmed these predictions by repeating the experiment with highly buffered media (Fig 4). Our results indicate that density-dependence can have a marked effect on growth dynamics, even in a simple constant drug setting.

### Density effects mitigated by time-dependent dosing strategies in a sub-MIC model of growth dynamics

Because density dependence can impact constant drug treatments, we next asked whether the efficacy of such drugs could be improved by judiciously dosing the drug over time. To answer this question, we again consider a simplified scenario where a logistically growing population is treated with sub-MIC concentrations of a drug that exhibits density dependent efficacy (measured again by ε). To be conservative, we restrict ourselves to populations growing in the approximate density regime we measured experimentally; specifically, we consider a population with a starting density of OD = 0.1—just below the densities measured—and limit growth by introducing a carrying capacity of C = 1.3, just below that measured in our culture devices (Fig 1D, inset). For simplicity, we do not consider clinically relevant periodic dosing schedules or super-MIC dosing regimens in this section, but we develop a more realistic model in a later section.

Using this sub-MIC growth model, we compared a naïve dosing strategy where drug is added at a concentration D_0_ at time t = 0 to an optimal, step-like dosing protocol where drug is added at concentration (D_0_/τ) at time t = 0 and then “switched off” (set to 0) at time τT, with τ (0≤τ≤1) chosen to minimize the total population density n(T) at the end of the treatment period T (see Methods). In the absence of density dependence, both dosing strategies yield a time-averaged drug concentration $\langle D\rangle =\frac{1}{T}\int_{0}^{T}D(t)dt=D_{0}$. Note that D(t) is the external concentration of drug at time t in the absence of density-dependent concentration changes; for simplicity, we do not consider the flow dynamics needed to establish and maintain such a concentration, though these details could be readily incorporated for any particular flow system.

When a drug exhibits density-dependent inhibition (ε>0), the two dosing protocols often yield significantly different results. Specifically, the optimal step-like dosing protocol can decrease the final population size by more than 25% relative to the naïve protocol, depending on the strength of density dependence (ε) and the drug concentration <D> (Fig 4B). For example, in the case of tigecycline (ε = 0.9), the optimal step-like dosing protocol decreases population size by more than 10% when D_0_/K_0_ ≈ 1 (Fig 4B, main panel: dashed line and inset). These differences arise because the effective drug concentration decreases dramatically in growing populations due to density-dependent inhibition, rendering late-stages of the therapy less potent than expected (Fig 4B, upper right inset). Our results suggest that even when density-dependent inhibition limits the efficacy of a given drug, an optimal step-like dosing strategy can mitigate the density dependence and reduce its impact (Fig 4B, bottom right inset).

The step-like protocol—which employs higher drug concentrations when drug is most effective—is reminiscent of the response-time strategy proposed to deal with cells expressing β-lactamase [8], though here we derive it from experimental measurements and a phenomenological model, not a mechanistic model. The benefit of optimal treatment is typically maximized at <D> ≈ K_0_, suggesting that this strategy may be useful when drug is limited because of cost, toxicity, acquired resistance, or delivery limitations. On the other hand, when D_0_ >> K_0_, optimal dosing does not have a significant effect on the total population size because nearly complete inhibition can be achieved with either protocol. In the specific case of tigecycline, concentrations much larger than K_0_ should be achievable in vivo [39]. However, optimal dosing could be important after mutants acquire resistant or for other drugs with similar density dependence profiles. For example, *E*. *faecalis* V583 is more resistant to spectinomycin (K_0_ = 102±1 ug/mL), and K_0_ is the same order of magnitude as in vivo serum levels in some cases [40]. As a whole, these calculations show that treatment strategies can, in principle, be improved using time-dependent dosing when density-dependent efficacy is significant.

### Density dependence of antibiotic inhibition leads to bistable treatment outcomes in a pharmacokinetic / pharmacodynamic (PK/PD) model of infection

To gauge the impact of density dependence on more realistic treatment dynamics, we incorporated our measurements into a simple pharmacokinetic / pharmacodynamics (PK/PD) model of infection similar to those from [6, 41] (see Methods and S1 Text). Briefly, the model considers cyclic dosing of antibiotic with period T, leading to time-dependent effective drug concentration D(t) that starts at a maximum concentration D_0_ at the beginning of each dosing period. In the absence of density dependence, the concentration D(t) decays exponentially at a rate k_d_, which accounts for decay of drug in the clinical system of interest (e.g. a patient). To incorporate density dependence, we again incorporate an additional decay term (–εDn^j^) in the equation for D(t). Bacteria grow logistically and respond to drug according to a Hill-like pharmacodynamics function that ranges from maximum growth (g_max_, which we set equal to 1 without loss of generality) to a minimum growth characterized by a maximum rate of kill (g_min_ < 0). The concentration, K_0_, at which growth is zero is defined as the MIC (by analogy with the half-maximal inhibitory concentration of the in vitro model, we choose the same notation K_0_ to now represent the MIC). Because we have not measured the maximum kill rates (g_min_) for the drugs in this study—and because those rates may depend on specific environmental factors—we consider a wide range of kill rates both larger and smaller in magnitude than the native growth rate of the population.

**Fig 5:**
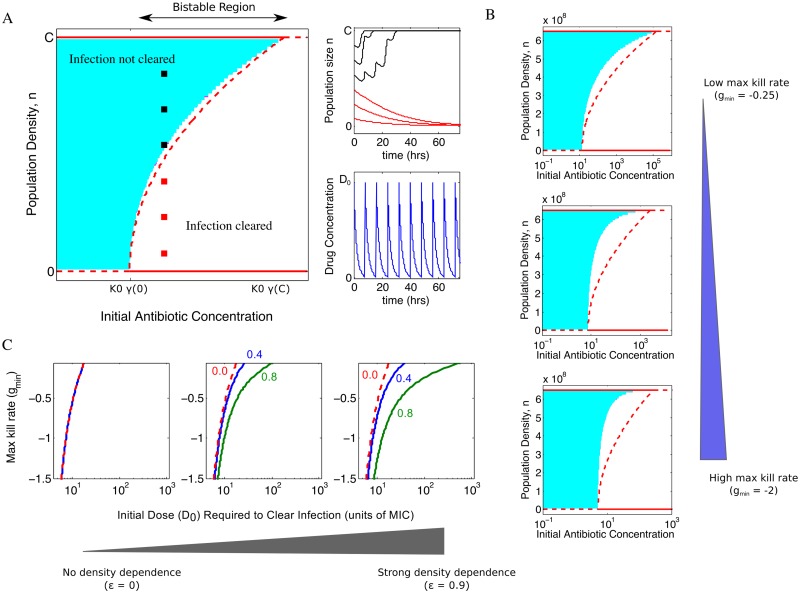
Density dependence of antibiotic leads to bistable treatment outcomes and potential treatment failure in a pharmacokinetic / pharmakodynamic (PK/PD) model of infection. **A.** Main panel**:** Theoretical (solid and dashed lines) and numerical (shaded region) phase diagrams indicate treatment outcomes in PK/PD model as a function of initial cell density (ranging from 0 to the carrying capacity, C) and initial antibiotic concentration D_0_. Solid red lines, stable fixed points of population density (theory). Dashed red lines, unstable fixed points (theory). The curved dashed red line is the phase boundary (separatrix) indicating the critical density above which a population will survive. A region of growth bistability, where treatment can lead to success or failure depending on initial cell density, exists for antibiotic concentrations *K*_0_*γ*(0) ≤ *D*_0_ ≤ *K*_0_*γ*(*C*), where K_0_ is the MIC and γ(n) is a nonlinear function that depends on the maximum drug kill rate (g_min_), the Hill coefficient (h), the drug decay rate (k_d_), and the dosing period T (S1 Text). Shaded regions indicate treatment failure in numerical solutions of the PK/PD model. Upper right inset: numerical solution of PK/PD equations for five different initial densities (indicated by red and black squares on the phase diagram). Lower inset: temporal dynamics of antibiotic concentration. For numerical phase diagram and simulations, g_min_ = -0.05 and ε = 0.9. Simulations in insets correspond to D_0_ = 200 (in units of MIC, K_0_). **B.** Phase diagrams from both theory (solid and dashed lines) and numerical simulations (shaded region) for increasing maximum kill rates (g_min_ = -0.25, -1, -2 from top to bottom) and populations densities on the order of 10^8^ cells/mL (corresponding to the OD ranges measured here). g_min_ is measured in units of g_max_; biologically, g_max_≈1 hr^-1^ for bacteria, so one can also view these units as inverse hours. **C.** Initial dose of antibiotic (units of MIC, K_0_) required to clear infections of density OD = 0 (dashed red), OD = 0.4 (blue line), and OD = 0.8 (red line) for different maximum kill rates for the case with no density dependence (ε = 0, left), modest density dependence (ε = 0.5, middle), and strong density dependence (ε = 0.9, right). In all panels, the Hill coefficient h = 2, k_d_ = ½, and T = 8, corresponding to a treatment period of 8 hours and a natural drug decay rate of ½ hr^-1^. Qualitatively similar results are found for other parameters (Figure G of S1 Text).

To study the effect of density-dependence on long-term treatment strategies, we analytically derived a full phase diagram (Fig 5A) describing the steady state behavior of the system as a function of initial drug dose D_0_ and population density n. The analytic calculations involve an adiabatic elimination of the fast timescale dynamics of D(t) (S1 Text) and are increasingly accurate in the limit of small maximum kill rate (|g_min_|<<1. We find that for sufficiently small D_0_, the infection cannot be cleared regardless of initial density. Similarly, for sufficiently large D_0_, the infection will always be cleared. Interestingly, however, there is a range of drug concentrations where the outcome is bistable and high-density infections cannot be cleared (S1 Text). Specifically, bistability occurs when ε>0 for initial drug concentrations in the range *K*_0_*γ*(0) < *D*_0_ < *K*_0_*γ*(*C*), with γ(n) a density-dependent function that depends on the strength of the density dependence (ε), the maximum kill rate (g_min_), the Hill coefficient (h), and the dosing protocol (specifically, the period T and the natural decay rate k_d_ of the drug). For drug concentrations in the bistable region, infections with a density greater than some critical value cannot be cleared.

These analytical approximations qualitatively describe numerical solutions of the model (Fig 5). For numerical analysis, we take T = 8 hours and k_d_ = 0.5 hr^-1^ (as in [6, 41]), though similar qualitative results are obtained for a range of parameters (see Figure G in S1 Text). In addition, for drugs whose efficacy increases with density (ε<0, such as for ampicillin), bistability cannot exist. Instead, the non-trivial fixed point becomes stable, leading to a drug-dependent stable population size (Figure G in S1 Text). In that case, there is an intermediate range of drug concentrations for which population size will either increase or decrease from its initial value depending on the specific location in the phase diagram.

Overall, the analytical approximation accurately predicts the entire phase diagram when the drug is characterized by a small kill rate (Fig 5A) but systematically underestimates the critical density for large kill rates (Fig 5B). This underestimation arises, in part, because solutions that might decay in the long-time limit go extinct following a small number of doses when the maximum kill rate is large. In addition, large kill rates mean that n(t) is often changing on a timescale similar to that of D(t), precluding the separation of timescales required for an accurate adiabatic approximation.

To further explore these density-drive treatment effects, we numerically calculated the initial dose D_0_ required to clear infections at various densities (Fig 5C). When there is no density dependence (left), bistability is absent and infections are cleared for initial doses ranging from 5–20 times the MIC, depending on the kill rate of the drug. On the other hand, when modest (middle) or strong (right) density dependence exists—similar in size to the effects measured in this study—the treatment is bistable for a large range of concentrations, indicating that initial dosages hundreds or even thousands of times larger than the MIC can fail to clear particularly dense infections (OD≈0.8), especially when maximum kill rate is relatively small. Overall, our numerical and analytical results indicate that density-dependent drug efficacy can lead to bistability in treatment outcomes over a broad range of parameters, indicating that successful treatments based on traditional (low density) MIC measurements may often fail when applied to sufficiently dense populations.

## Discussion

Using computer-automated turbidostats, we have directly measured the effects of population density on antibiotic inhibition in *E*. *faecalis*. In contrast with traditional measurements of the IE, which provide only a binary measure (survival, death) of density dependence, here we provide unambiguous, fixed-density measurements of per capita growth rate as a function of density and drug concentration. Using this functional relationship—which is quantitatively described by a phenomenological rate parameter ε—we are able to optimize time-dependent drug dosing protocols, including a clinically-inspired pharmacology model that involves periods of both super- and sub-MIC drug exposure. More specifically, we show that density-dependence can significantly impact growth dynamics in constant drug environments, while time-dependent dosing strategies can partially mitigate these effects. Perhaps most strikingly, the observed density dependence can induce bistability of treatment outcomes over a wide range of antibiotic doses—in some cases, more than 1000 times the MIC of the drug—rendering otherwise successful protocols potentially ineffective for the treatment of dense populations. We analytically derive an expression for the size of the bistable region, and our analysis allows us to calculate the critical population density for treatment failure using common pharmacological parameters.

Our results have implications for both basic and applied biology. From a basic science perspective, they underscore the notion that bacterial physiology cannot be cleanly divided into three growth phases: lag phase, exponential phase, and stationary phase. Instead, our measurements show that substantial changes in growth dynamics occur when bacteria are under stress, even in relatively narrow density ranges in exponential phase. In addition, we’ve shown that density-driven changes in pH modulate inhibition for some drugs. It is surprising that (to our knowledge) pH not been previously linked with the IE, because pH is known to modulate drug efficacy and to depend on bacterial growth phase. However, classical IE measurements involve standardizing only the initial pH of the media, but like density, that pH may change significantly during the experiment, perhaps obscuring any pH effects. Finally, in contrast to the classical inoculum effect, we found that the inhibitory effects of some drugs, such as ampicillin, increase at high densities. Overall, these findings represent a new entry in a growing catalog of density-mediated physiological changes in bacteria, including modulation of metabolism [42], quorum sensing [43], and synchronization of gene expression [44]. While it is well known that bacteria in different growth phases may respond differently to antibiotics—with biofilms [45, 46] and dormant persister cells [47] representing two of the most salient examples—it is notable that such significant density-dependent effects can also arise in exponential phase, where growth in the absence of drug is approximately constant. On a practical level, our findings may offer a first step toward systematic optimization of antimicrobial-therapy based on population density. In addition, they may be important for industrial applications—including microbial fermentation [48]—where optimizing growth may also require consideration of density-mediated changes in population dynamics.

However, it is important to keep in mind several limitations of our study. First, aside from the pH-mediated effects that explain a fraction of our results, we have not attempted to elucidate the molecular mechanisms responsible for the observed density dependence. Indeed, as suggested by other studies, it seems likely that such dependence—and the IE in general—results from a combination of factors and could differ for each drug [2–6]. For that reason, we focused instead on the functional implications of our measurements for population growth and drug response. Secondly, we measured population density using light scattering, which is a widely used method for inferring cell density but could conceivably conflate changes in cell number with changes in cell shape. Because growth rates are calculated from pump flow rates required to maintain constant light scattering intensity over a narrow range—not directly from time series of optical density—we do not anticipate significant artifacts from this limitation. We’ve also restricted our measurements to sub-MIC levels of antibiotics, where cell filamentation is minimal. Nevertheless, one should interpret the specific functional forms of the density dependence with some caution, particularly for drugs such as ciprofloxacin that can induce filamentation, as optical density may not be strictly proportional to viable cell number at high drug concentrations. In addition, the sensitivity of light scattering limits our measurements to relatively dense populations (OD>0.1), though measurements of the inoculum effect suggest that density-dependence may span several orders of magnitude [1, 4, 9]. It is not clear whether the density dependencies we observe may extend to lower densities. If so, that would dramatically increase the effect of density-dependence on time-to-threshold measurements, optimal dosing strategies, and PK/PD-based treatment protocols.

We also stress that applying results from in vitro measurements to realistic in vivo scenarios is a significant challenge. In an attempt to emphasize density-dependent effects in a transparent setting, we incorporated our results into both a simple model for sub-MIC growth dynamics as well as a PK/PD model for antibiotic treatment. However, we acknowledge that these models neglect important factors such as the response of the host immune system [49] as well as other PD phenomena, including the post-antibiotic effect [50], that may impact in-vivo treatments. Despite these caveats, our findings provide experimental and theoretical evidence that antibiotic efficacy depends dramatically on population density, and this dependence may, in turn, dictate the success or failure of standard treatment protocols. Bistability may be a particularly important consideration for drugs with low kill rates, and many drugs exhibit in-vitro kill rates on the lower end of the range considered here when applied to *E*. *faecalis* [51–53]. More generally, because our results are based on phenotypic measurements, they allow for optimization and prediction of drug dosing protocols even when molecular mechanisms are not fully known. Such mechanism-free phenomenological approaches are promising because of their potential applicability in more realistic clinical scenarios, where phenotypic measurements—for example, an estimate of the parameter ε—may be tractable even without mechanistic information [8, 36, 54–59]. Our hope is that these findings motivate continued quantitative studies on the IE and lay the groundwork for more detailed clinical models.

Finally, our results also raise a series of fundamental questions at the interface of clinical, basic, and evolutionary microbiology. Perhaps most interestingly, the density dependence of antibiotic efficacy suggests that the relative fitness of drug-resistant cells may be intricately linked with the total density of the surrounding microbial population. As a result, the dynamics of evolving populations may exhibit many of the complex spatial and temporal dynamics described by evolutionary game theory [38], even beyond the known cases that depend on enzymatic drug degradation [3, 9]. At a practical level, these results also raise fundamental questions about the impact of antibiotic therapy on the evolution of drug resistance. In the long run, effective antimicrobial therapies must balance the inhibitory effects of treatment with its propensity to promote drug resistance, a topic of considerable recent interest [60, 61]. Our results suggest that both of these factors could depend significantly on cell density, and we hope our work motivates continued efforts to understand the potentially complex interplay between population density, optimal treatments, and the evolutionary dynamics of resistance.

## Methods

### Bacterial strains, media and growth conditions

All experiments were performed with *Enterococcus faecalis* V583, a fully sequenced vancomycin-resistant clinical isolate [20]. Cultures for experiments were taken from single colonies grown on plates of BHI agar and then cells were grown at 30°C in sterile Brain-Heart Infusion media (Remel) overnight before being diluted in fresh media 500X prior to experiment start. Experiments with pH-stabilized media had cells suspended in BHI supplemented with 50 mM Sodium Phosphate (Fisher).

### Drugs

Antibiotics used in the study include Ampicillin Sodium Salt and Doxycycline Hydrochloride (Fisher); Ciprofloxacin and Ceftriaxone Sodium Salt (Acros Organics); Nitrofurantoin and Spectinomycin Sulfate (MP Biomedicals); Tigecycline (TSZ Chem); Linezolid (Chem-Impex Int’l LLC); and Daptomycin (Fisher). See Table A in S1 Text.

### Continuous Culture Devices

We constructed customized continuous culture devices based on similar designs from several recent works [14–16]. In particular, the in-depth protocol from [16] was instrumental in our design process and is highly recommended to others looking to build customized instruments. Briefly, in our instrument, an infrared LED emitter (Radioshack) shines through the side of a flat bottom glass vial positioned on a multi-position magnetic stir plate. IR light passes through the culture solution (approximately 15 mL) and is scattered onto a photodiode detector (Radioshack) at an angle of approximately 130 degrees from the emitter. Photodiode voltage is directly proportional to the optical density of cells inside the vial. Populations are held at designated densities via custom MATLAB scripts, built on the Matlab Instrument Control Toolbox, which control a series of peristaltic pumps (Boxer 15000, Clark Solutions) via multi-channel analog output device (Measurement Computing USB-3103 and USB-3105), which control pump speed, and a relay board (Measurement Computing USB ERB-24) to allow us to turn pumps on or off. Voltages from up to 18 vials are simultaneously recorded from 18 photodiodes via a series of analog input boards (Measurement Computing USB 1616FS). When the voltage is above a threshold value, a signal is sent to the relay board to turn on two pumps for that vial: one to introduce new media and the other to remove media and cells to keep vial volume constant. When voltage returns below a set value through dilution, the pumps are turned off. The CCD and all tubing is thoroughly cleaned with bleach and de-ionized water after each experiment to prevent contamination.

### Constant Density Measurements

At the onset of the experiment, overnight stationary phase bacterial cultures are diluted 500X and allowed to grow in the culture vials until a specific density is reached. At that point, drug is manually added at the desired concentration to both the culture vial and a connected chamber with fresh media. Drug concentration is the same in both the culture vial and the fresh media vial. Flow between the media chamber, the culture vial, and a waste vial is managed by a series of computer-controlled peristaltic pumps that maintain constant cell density (Fig 1).

### Real-time and Steady-State Growth Rate Measurements

In our constant density turbidostat, the per capita growth rate, g, is equal to the relative dilution rate *g* = *F/V*, where F is the (potentially time-dependent) flow rate of the pumps and V is the total culture volume. We determined population growth rate by first setting the variable flow rate of each pump to a constant that we call F_max_ (F_max_ ≈ 1 mL/min). We then switched the pumps “on” or “off” to maintain constant cell density according to the scheme in Fig 1A. To estimate population growth, we measured the state of each pump (“on” or “off”) at each time step (Δt ≈ 1.5 seconds) during this constant density phase. The fraction of time the pump is on, f_on_, is given by the number of time steps N_on_ for which the pump was “on” divided by the total number of timesteps (N_Total_) during a period of ~1–3 hours once population growth had reached a steady state. Flow rate F is related to f_on_ according to F = f_on_ F_max_, and hence growth is given by $g=\frac{f_{on}F_{max}}{V}$. To verify that the growth was in steady state, we estimated real time growth rate (Fig 1C) using a moving average of the relative dilution rate (fraction of time in “on” state) over a window size of approximately 15 minutes. All values are relative to growth rates measured in the same vial and at the same density in the absence of drug (doubling time of approximately 30–35 minutes).

### Statistical Analysis

We consider two sources of experimental uncertainty in our measurements. First, there is uncertainty in the measurement of steady state growth rate in a single vial. The growth rate is given by $g=\frac{f_{on}F_{max}}{V}$, where f_on_ is the fraction of time the pump is “on”, F_max_ is the maximum flow rate of the pump, and V is the volume of the culture. F_max_ and V are assumed to be known exactly for a given vial, so the uncertainty comes from the measurement of f_on_. This uncertainty depends on the length of time over which growth rate is measured: very long measurements give an increasingly accurate picture of the growth rate. In steady state growth, we expect the uncertainty in the number of time steps for which the pump is on to be approximately (N_on_)^1/2^, so the fraction of time the pump is on is given by $f_{on}=\frac{N_{on}}{N_{Total}}\pm \frac{\sqrt{N_{on}}}{N_{Total}}$, where N_Total_ is the total number of time steps over which growth is measured (and is known exactly). To estimate the relative per capita growth, we calculated f_on_ with and without drug in the same vial and at the same density. The uncertainty in the ratio between f_on_ with drug and f_on_ without drug is then given by standard error propagation for a ratio of two variables. Specifically, the uncertainty in the ratio z of two uncorrelated variables x and y is given by $\delta z=\left|z\right|\sqrt{{\left(\delta x/x\right)}^{2}+{\left(\delta y/y\right)}^{2}}$.

The second source of variability comes from vial-to-vial variations. To account for these variations, we measured relative growth in duplicate or triplicate for each condition. The final growth rate measurement is the mean over these trials. Because each trial has an associated uncertainty, the total uncertainty is given by propagating the uncertainty from each individual measurement into a single uncertainty for the mean. This calculation draws on standard error propagation for sums of random variables. Specifically, for a sum of two uncorrelated random variables z = x+y, the uncertainty is given by $\delta z=\sqrt{\delta x^{2}+\delta y^{2}}$.

From a growth measurement g with uncertainty δg, 95% confidence intervals are taken to be *g* ± 1.96 *δg*, which essentially assumes (by the Central Limit Theorem) that the total measurement noise is Gaussian. Measurement differences are deemed statistically significant if and only if 95% confidence intervals do not overlap (this is a somewhat conservative estimate, as differences can be statistically significant even when confidence intervals do overlap, though the converse is never true; here we use the more stringent criteria of non-overlapping confidence intervals).

### Mathematical model of turbidostat growth and estimation of rate constant ε

We modeled density dependent drug inhibition in the turbidostat by assuming that the effective drug concentration D in each vial changes according to $\dot{D}=\left(F/V\right)\left(D_{in}-D\right)-\varepsilon Dn^{j}$, where F is the flow rate of the pumps, V is the volume of media in the culture chamber, D_in_ is the drug concentration in the feed chamber (measured in units of K_0_), and the last term (-εDn^j^) accounts for density-dependent effects on drug inhibition (we restrict ourselves to models with j = 1 or j = 2). The per capita growth g (measured in units of growth rate in the absence of drug) is related to the pump flow rate (g = F/V) and also to the effective drug concentration in the vial ($g\left(D\right)={\left(1+{\left(D/K_{0}\right)}^{h}\right)}^{-1}$). Therefore, the steady state behavior is given by ${\left(\frac{1-g}{g}\right)}^{1/h}=\frac{D_{in}}{1+\varepsilon n^{j}(g^{-1})}$, which can be solved numerically to give g for any combination of D_in_, h, j, and ε. We use this model along with standard model selection techniques [62] to estimate ε and determine the best decay model (j = 1 or j = 2) for each drug directly from turbidostat data (S1 Text, including Table B and Figures E and F).

### Mathematical model for population growth and parameter estimation

We modeled density dependent drug inhibition by extending a classic logistic model of bacteria growth to include density- and drug-dependent population growth. Specifically, we have

$$\dot{n}=g\left(D\right)\left(1-\frac{n}{C}\right)n$$ (1)

$$\dot{D}=-\varepsilon Dn^{j}$$ (2)

where n is the cell density, C is the carrying capacity of the environment, the factor of (1 − *n*/*C*) corresponds to linear decrease in growth rate as n approaches the environmental carrying capacity, and g(D) is the per capita growth rate of the population exposed to drug at effective concentration D. Specifically, we have $g\left(D\right)={\left(1+{\left(D/K_{0}\right)}^{h}\right)}^{-1}$, with K_0_ the IC_50_ of the drug and h a Hill-like steepness coefficient, similar to dose response models in pharmacology [34]. To model the observed density dependence, we allow the effective drug concentration D to decrease (ε>0) or increase (ε<0) proportionally to Dn^j^, where j = 1 or j = 2 to indicate linear or quadratic decay with density, respectively (Eq 2). However, we stress that more general forms of drug decay may be better suited to some drugs. We choose the form above as a simple analytical parameterization that captures the primary features of our measurements (Figure D of S1 Text). Alternatively, in cases where mechanistic understanding is available, the form of the decay may potentially be derived from a microscopic model (for example, a linear function of n may be appropriate to describe density effects that arise from enzyme production). We also stress that K_0_ and h can be estimated from traditional growth curves, and only the parameter ε is estimated from our constant-density measurements.

### Optimal Step-Like Dosing

To investigate the effects of dosing schedule on sub-MIC growth dynamics, we compared total population size n(T) at the end of a treatment of length T for cells exposed to two different dosing protocols. In both cases, the dynamics are given by Eqs 1 and 2, and T is chosen to correspond to the approximate time the population needs to reach carrying capacity in the absence of drug. In the absence of density dependence, both protocols yield an average effective drug concentration of $\langle D\rangle =\frac{1}{T}\int_{0}^{T}D\left(t\right)\;dt=D_{0}$.

1. Naïve Protocol: The effective drug concentration is set to D_0_ at time 0 and the system evolves for total time T.
2. Step-Like Optimal Protocol: The effect drug concentration is set to D_0_/τ at time 0 and the system evolves for a time τT, with 0≤τ≤1. Then, at time τT, the drug concentration is set to 0 and the system evolves to a final time T. We choose τ to numerically minimize n(T)—the final population size—for each value of D_0_.

### PK/PD Model for infection dynamics

Please see S1 Text for a detailed description of the PK/PD model as well as the derivation of the phase diagram for treatment bistability. Briefly, the PK/PD model describes antibiotic treatment that is dosed periodically with period T; at the beginning of each period, the effective drug concentration is set to D_0_. During the dosing period, the drug decays according to Eq 2 (due to density dependent inhibition), but we also include an additional linear decay (with a rate k_d_) to account for natural (density independent) drug decay in clinical setting. The effective concentration is then reset to D_0_ at the next cycle. The cell density n changes with instantaneous D(t) according to Eq 1, but g(D) is now a monotonically decreasing function of D that crosses g(D) = 0 at D = K_0_, where K_0_ is defined as the MIC value, and asymptotically approaches some maximum kill rate (g_min_<0). The model can be easily solved numerically using any ODE integration software (we used Matlab’s ode45 function). To study the long-time behavior of the system, we assumed n(t) is approximately constant on the timescale of D(t). We then averaged the dynamics of D(t) over one period, yielding an approximation equation for n valid in the limit of small g_min_. We then performed linear stability analysis on the fixed points of the averaged equation to analytically derive phase diagrams (S1 Text).

## Supporting Information

S1 Text The supplemental S1 Text contains expanded description of mathematical models, details of parameter estimation and model selection, and 7 supplemental figures + 2 supplemental tables. (PDF)

S1 Data Density-dependent growth rate data. (XLSX)
