# Supplementary material for: Population Density Modulates Drug Inhibition and Gives Rise to Potential Bistability of Treatment Outcomes for Bacterial Infections
Source: PLoS Comput Biol. 2016 Oct 20;12(10):e1005098. doi: 10.1371/journal.pcbi.1005098 (PMC5072716; doi:10.1371/journal.pcbi.1005098)
Supplement: S1 Text — (PDF) [file pcbi.1005098.s001.pdf]

Supplemental Text 1: Population density modulates drug inhibition and gives rise to potential bistability of treatment outcomes for bacterial infections

1 Modeling Density Dependence in Turbidostat

1.1 Mathematical Model of Turbidostat Growth

In the turbidostat, cell density n is held constant using feedback control with peristaltic pumps (see Methods). Once cells have grown to the appropriate density, drug is added at concentration D_{in} to both the media feed reservoir and the culture vial. The drug concentration D in the reservoir is then given by

$$\frac{\partial D}{\partial t} = \frac{F}{V}(D_{in} - D) + f(n, D), \tag{S1}$$

where F is the flow rate of the pumps required to maintain constant density, V is the volume of the culture chamber, and $f(n, D)$ is an unspecified function that accounts for the effective change in drug concentration due to cell density. Here we take $f(n, D) = -\epsilon D n^j$, where ϵ is a rate constant and j is an exponent that describes the kinetics of drug decay (i.e. $j = 1$ indicates decay that is linear in n, while $j = 2$ indicates quadratic decay). This form (with $j = 1$ or $j = 2$) provides a good description of the turbidostat data for the drugs in this study (Figures E and F in S1 Text).

To maintain constant cell density, the flow rate must be related to per capita growth rate g by $g = F/V$, and Equation S1 therefore becomes

$$\frac{\partial D}{\partial t} = g(D_{in} - D) - \epsilon D n^j, \tag{S2}$$

which has steady state solution

$$D = \frac{D_{in}}{1 + \frac{\epsilon n^j}{g}}. \tag{S3}$$

The growth rate g, which we measure in dimensionless units of per capita growth rate in the absence of drug, is also dependent on the steady state drug concen-

tration, D, according to the Hill-like dose response function

$$g = (1 + D^h)^{-1}, \tag{S4}$$

where h is a Hill coefficient and D is measured in units of IC_{50}. We can combine Equation S3 and Equation S4 to arrive at a single nonlinear equation for g (or equivalently, for D). Specifically, we have

$$\left(\frac{1-g}{g}\right)^{1/h} = \frac{D_{in}}{1 + \frac{\epsilon n^j}{g}}. \tag{S5}$$

Equation S5 can be solved numerically to find g for any given values of n, D_{in} and the parameters h, j, and ϵ. In principle, Equation S5 can have multiple solutions, but we do not find evidence for multiple solutions ($g \geq 0$) over the parameter range of our measurements. The parameter h can be determined from standard low-density dose response curves (for example, $h \approx 2.1$ for tigecylcine; see main text), and ϵ is a free parameter that we estimate using turbidostat data (see Table B in S1 Text).

1.2 Parameter Estimation and Model Selection

To statistically compare the the two models for drug decay ($j = 1$ or $j = 2$), we use standard model-selection techniques [68]. Specifically, we assume that the experimental errors are independent and Gaussian distributed with unknown variance σ^2. We then calculate for each model the Akaike Information Criteria, which is given by

$$\text{AIC} = -2\log(\mathcal{L}(\hat{c}|y)) + 2m \tag{S6}$$

where $\log(\mathcal{L}(\hat{c}|y))$ is the log likelihood function, y is the turbidostat growth data, c is maximum likelihood estimate of the free parameters of the model (in this case, the decay rate constant ϵ and, implicitly, the unknown error variance), and m is the number of free parameters ($m = 2$ for both models). Note that the maximum likelihood estimate is the same as the least squares estimate for the Gaussian noise case. The AIC is an estimate of the expectation value of the relative Kullback-Leibler (KL) divergence between the fitted model and the "true mechanism" generating the observed data. The model with the lowest AIC value among a set of models is considered the best model in that it minimizes the KL divergence between the model and statistical mechanism underlying the data. For independent Gaussian errors, AIC reduces (up to an additive constant) to

$$\text{AIC} = -N\log(\hat{\sigma}^2) + 2m, \tag{S7}$$

where N is the number of observations and $\hat{\sigma}^2$ is the maximum likelihood estimate of the variance. In practice, we use a small sample estimator of AIC that includes a bias correction term

$$\text{AIC} = -2\log(\mathcal{L}(\hat{c}|y)) + 2m + \frac{2m(m+1)}{N-m-1}. \tag{S8}$$

The differences in AIC values between the two models can be converted to an Akaiki weight,

$$w = \frac{\exp(-\delta/2)}{\exp(-\delta/2) + 1} \tag{S9}$$

where $\delta \equiv \text{AIC}_2 - \text{AIC}_1$, where AIC_j is the AIC of model j. Because $\exp(-\delta/2)$ is proportional to the likelihood of the model j given the data, the weight w can be interpreted as a measure of the evidence in favor of one model or the other. Parameter estimates, AIC values, and weights for each model are given in Table SB.

2 PK/PD Model for Infection Dynamics

2.1 Model Definition

To model infection dynamics, we assume a simple PK/PD model similar to those in [6, 41]. Specifically, we assume cell density n and effective antibiotic concentration D are governed by

$$\begin{aligned} \frac{\partial n}{\partial t} &= g(D)\left(1 - \frac{n}{C}\right)n, \\ \frac{\partial D}{\partial t} &= -k_d D - \epsilon D n^j, \end{aligned} \tag{S10}$$

where $g(D)$ is a growth rate function that accounts for the effects of antibiotic, C is the carrying capacity of the environment, and k_d is the natural decay rate of the antibiotic in the clinical setting of interest (e.g. in the bloodstream of the patient). To account for the measured density dependence of drug efficacy, the equation for D involves a density dependent decay term similar to Equation S2. The growth rate function $g(D)$ describes the impact of drug on division rate and is given by [6, 41]

$$g(D) \equiv \frac{g_{max}\left(\left(\frac{D}{K_0}\right)^h - 1\right)}{\frac{g_{max}}{g_{min}}\left(\frac{D}{K_0}\right)^h - 1}, \tag{S11}$$

where g_{max} is the maximum growth rate of the population (which we can set equal to 1 without loss of generality by measuring time in units of g_{max}^{-1}), $g_{min} < 0$ is the maximum rate of kill of the drug, h is a Hill-like steepness coefficient, and K_0 is the minimal inhibitory concentration (MIC) of the drug. The growth function, Equation S11, is a monotonically decreasing sigmoidal function that equals zero at $D = K_0$ and asymptotically approaches g_{min} for high drug concentrations.

2.2 Periodic Drug Dosing

To mimic clinical dosing protocols [6, 41], we assume that drug is added at a concentration D_0 at periodic intervals with length T. In what follows, we outline

an adiabatic approximation to this model valid when the dynamics of $D(t)$ occur on a faster timescale than those for $n(t)$. This approximation provides a qualitative description of the system dynamics and is quantitatively accurate when $|g_{min}| << 1$.

2.2.1 Adiabatic Approximation

To make analytical progress, we assume that $n(t)$ changes slowly on the timescale of $D(t)$. As a result, the drug decays exponentially over each period according to

$$D(t) = D_0 e^{-k(n)t}, \tag{S12}$$

where $k(n) \equiv k_d - \epsilon n^j$, and $D(t)$ is reset to D_0 at the start of the next dosing period. Because we are interested in the long-time behavior of the system spanning many periods T, we approximate the dynamics of Equation S10 by inserting Equation S12 into Equation S10 for $\frac{\partial n}{\partial t}$ and then averaging over one period to arrive at

$$\frac{\partial n}{\partial t} = \langle g(n) \rangle \left(1 - \frac{n}{C}\right) n, \tag{S13}$$

where brackets represent an average over one period T. This amounts to an adiabatic approximation that averages over dynamics on the faster timescale governing $D(t)$. We expect this approximation to be valid when the system requires multiple periods T to reach its steady state ($n = 0$ or $n = C$), and we find that it is increasingly accurate when $|g_{min}| << 1$, which achieves the desired separation of timescales. Specifically, we have

$$\langle g(n) \rangle = g_{min} + \frac{2(1-g_{min}) \tanh^{-1}\left(\frac{e^{\beta(n)h}-1}{1+e^{\beta(n)h}-\frac{2}{g_{min}}\left(\frac{D_0}{K_0}\right)^h}\right)}{\beta(n)h}, \tag{S14}$$

where $\beta(n) \equiv k(n)T$. Equation S14 follows from straightforward integration of the growth function over one period followed by simple algebraic manipulation (note that this form is valid only for $g_{min} < 0$, but the general case can be written in terms of logarithms rather than the $\tanh^{-1}$ function).

2.2.2 Fixed Points and Linear Stability

Using Equations S13, S14, one can show that the system has three fixed points (n_0, n_1, n_2) given by

$$\begin{aligned} n_0 &= 0, \\ D_0 &= K_0 \gamma(n_1), \\ n_2 &= C, \end{aligned} \tag{S15}$$

where $\gamma(n)$ is given by

$$\gamma(n) \equiv \left(\frac{g_{min}}{2}\left(1 + e^{\beta(n)h} + \left(e^{\beta(n)h} - 1\right)\coth\left(\frac{g_{min}\beta(n)h}{2(1-g_{min})}\right)\right)\right)^{1/h}. \tag{S16}$$

The fixed points occur when the population is extinct (n_0) or has reached carrying capacity (n_2). However, the density-dependence of the MIC gives rise to a third non-trivial fixed point (n_1) given by the (physically relevant) solutions to $D_0 = K_0\gamma(n)$, which ensure that $\langle g(n) \rangle = 0$. By linearizing Equation S13 around the fixed points in Equation 15, it is straightforward to show that n_0 is stable for $D_0 > K_0\gamma(0)$ and unstable for $D_0 < K_0\gamma(0)$. Similarly, fixed point n_2 is stable for $D_0 < K_0\gamma(C)$ and unstable for $D_0 > K_0\gamma(C)$. Finally, the physically meaningful values of the nontrivial fixed point n_1 are unstable for $\epsilon > 0$. On the other hand, if $\epsilon < 0$, the non-trivial fixed point becomes stable over the physically relevant range.

2.2.3 Bistability

Interestingly, this stability analysis points to a region of bistability for $K_0\gamma(0) < D_0 < K_0\gamma(C)$. The size of the bistable region is given by $\Delta D = K_0(\gamma(C) - \gamma(0))$. Because $\gamma(n) \sim \exp(\beta(n))$ for large n, the range of bistability can be extremely large, often many orders of magnitude larger than K_0 (see Figure 5). Examples of the bifurcation diagram for different values of g_{min} and $k_{1/2}$ are shown in Figure 5 and Figure G in S1 Text. Finally, we note that bistability cannot occur when $\epsilon < 0$, as for ampicillin. Instead, there is an intermediate range of drug concentrations for which all initial densities approach an intermediate, stable population size (Figure G in S1 Text).

References

[6] Udekwu, K.I., et al., *Functional relationship between bacterial cell density and the efficacy of antibiotics*. Journal of Antimicrobial Chemotherapy, 2009. 63(4): p. 745-757.

[41] Regoes RR, Wiuff C, Zappala RM et al. *Pharmacodynamic functions: a multiparameter approach to the design of antibiotic treatment regimens.* Antimicrob Agents Chemother 2004; 48: 3670-6.

[62] Burnham, K and Anderson, D (1998). *Model Selection and Multimodel Inference*, 2nd Edition, Springer-Verlag, New York.

Figures and Tables for S1 Text

Supplemental Figures and Table

Drug Name	Drug Class	Mechanism
Spectinomycin	Aminoglycoside	Interrupts protein synthesis
Linezolid	Oxazolidinone	Protein synthesis initiation inhibition
Doxycycline	Tetracycline	Blocks tRNA binding at ribosome
Ceftriaxone	Cephalosporin	Inhibits cell-wall synthesis by binding to transpeptidases
Tigecycline	Glycylcycline	Blocks animoacyl-tRNA interaction with ribosome
Nitrofurantoin	Unique Class	Reduced molecule is highly reactive and damages many biomolecules
Daptomycin	Lipopeptide	Aggregation in membranes leads to ion leakage from cells
Ampicillin	Beta-Lactam	Inhibits cell-wall synthesis by binding to transpeptidase
Ciprofloxacin	Fluoroquinilone	Stops cell division via inhibition of DNA gyrase and topoisomerase

Table A in S1 Text: Drugs Used in this Study.

	Linear Decay Model			Quadratic Decay Model		
Drug	**ε**	**AIC**	**Weight**	**ε**	**AIC**	**Weight**
**Tigecycline**	0.53±0.07	-90.0	0.02	**0.90±0.10**	**-97.0**	**0.98**
**Spectinomycin**	0.61±0.27	-83.6	0.08	**0.58±0.18**	**-88.6**	**0.92**
**Daptomycin**	**0.28±0.09**	**-73.2**	**0.91**	0.29±0.09	-68.5	0.09
**Nitrofurantoin**	8.00±12.00	-53.6	0.04	**5.50±2.51**	**-60.0**	**0.96**
**Ciprofloxacin**	0.81±0.33	-83.5	0.02	**0.77±0.20**	**-91.1**	**0.98**
**Linezolid**	**0.58±0.47**	**-84.9**	**0.53**	0.45±0.30	-84.6	0.47
**Ampicillin**	-0.13±0.40	-65.4	0.05	**-0.27±0.19**	**-71.1**	**0.95**
**Ceftriaxone**	0.02±0.19	-105.4	0.50	0.02±0.18	-105.4	0.50
**Doxycycline**	**0.27±0.09**	**-111.9**	**0.95**	0.20±0.08	-105.8	0.05

Table B in S1 Text: Parameters, error estimates, and Model Selection. The model highlighted in bold for each drug is the model selected according to AIC. Parameter values are shown with +/- one standard error of the fitted parameter. The units of ε are $([\text{time}][\text{cell density}]^{j})^{-1}$, where time is measured in units of inverse per capita growth rate, and cell density is measured in units of OD.

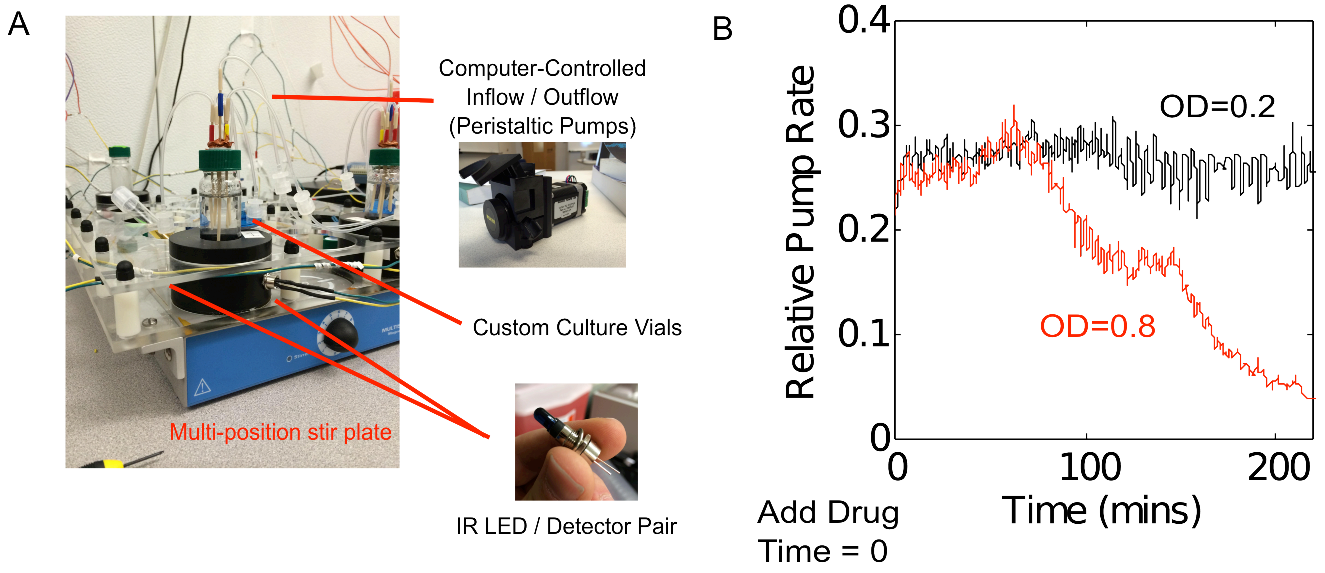

Figure A in S1 Text: Continuous culture devices and ampicillin growth. A. Bacterial cultures (15 mL) are grown in glass vials with customized Teflon tops that allow inflow and outflow of fluid via silicone tubing. Flow is managed by a series of computer-controlled peristaltic pumps—up to 6 per vial—which allow precise control of multiple environmental conditions (media, drug and nutrient concentration, waste removal). Cell density is monitored by light scattering using infrared LED/Detector pairs on the side of each vial holder. Up to 18 cultures can be grown simultaneously using a multi-position magnetic stirrer. The entire system is controlled by custom Matlab software, which manages pump flow to maintain constant density. **B.** Population growth rate in response to ampicillin (300 ng/mL) does not reach steady state on the timescale of the experiment. However, there are still clear differences between growth in low density cultures (black) and high density cultures (red) in exponential phase. For ampicillin, growth rate represents a time-averaged growth between 100-200 minutes after drug exposure.

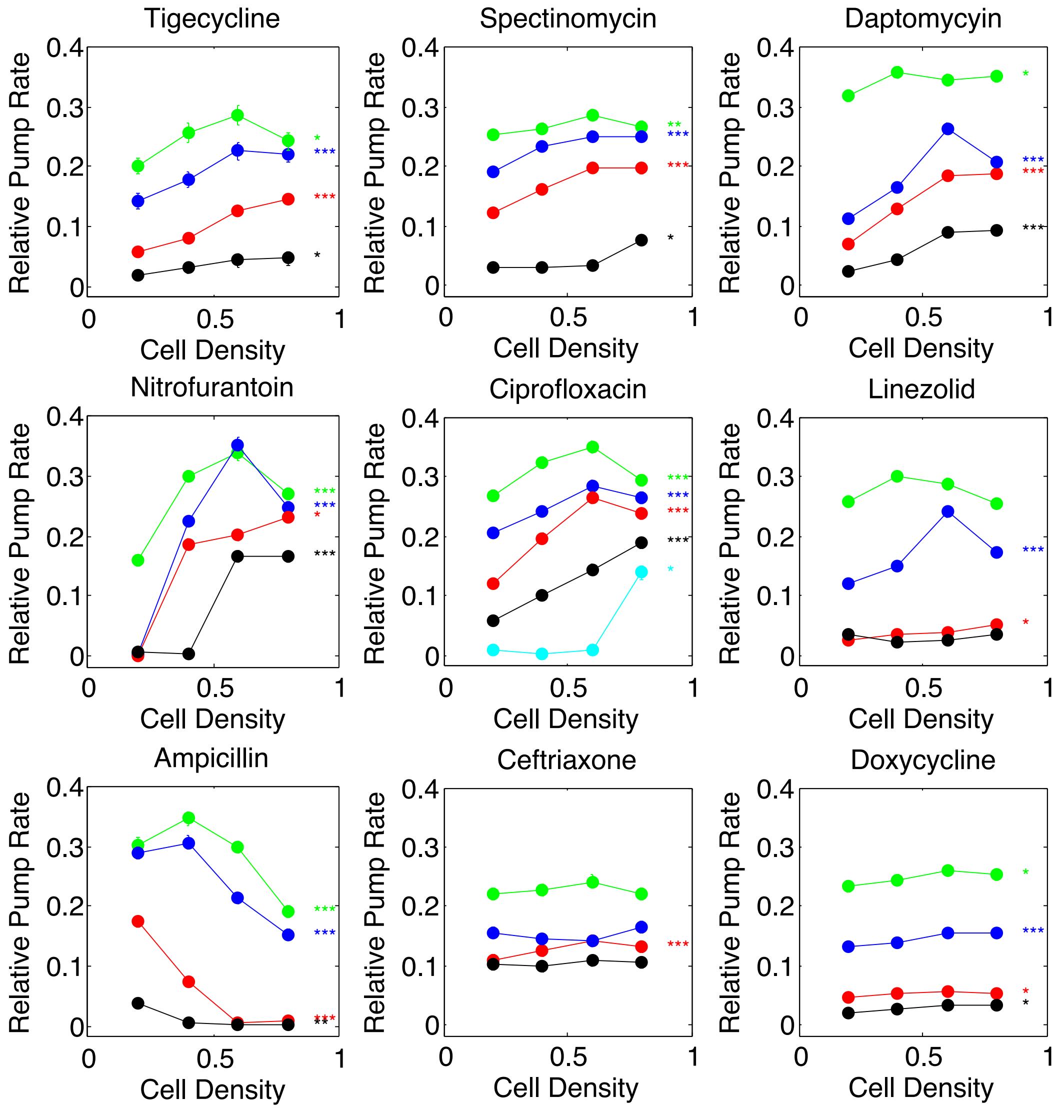

Figure B in S1 Text: Average pump rate (relative to maximum value) as a function of cell density. This figure is identical to Figure 1, but without the normalization from measurements under the same conditions but without drug. **A-I:** Steady state pump flow rate (relative to maximum pump rate, when the pumps are always on) as a function of cell density for multiple drug concentrations. Drug concentrations are **A.** Tigecycline concentration = 15 (green), 25 (blue), 50 (red), 100 (black) ng/mL; **B.** Spectinomycin concentration = 50 (green), 100 (blue), 150 (red), 400 (black) µg/mL; **C.** Daptomycin concentration = 1.0 (green), 1.25 (blue), 1.50 (red), 3.0 (black) µg/mL; **D.** Nitrofurantoin concentration = 50 (green), 100 (blue), 125 (red), 250 (black) µg/mL; **E.** Ciprofloxacin concentration = 100 (green), 150 (blue), 200 (red), 300 (black), 400 (cyan) ng/mL; **F.** Linezolid concentration = 0.1 (green), 0.5 (blue), 4 (red), 5 (black) µg/mL; **G.** Ampicillin concentration = 200 (green), 300 (blue), 400 (red), 500 (black) ng/mL; Note that ampicillin growth does not reach steady state on the time-scale of our experiment, so these measurements are effective growth rates averaged over a non-steady state (Figure S1). **H.** Ceftriaxone concentration = 5 (green), 50 (blue), 200 (red), 300 (black) µg/mL; **I.** Doxycycline concentration = 33 (green), 100 (blue), 333 (red), 500 (black) ng/mL. Statistically significant differences between growth at lowest and highest densities (0.2 and 0.8), intermediate densities (0.4 and 0.6), or both are indicated by *, **, and ***, respectively. Error bars are +/- 1.96 standard error (95% confidence intervals).

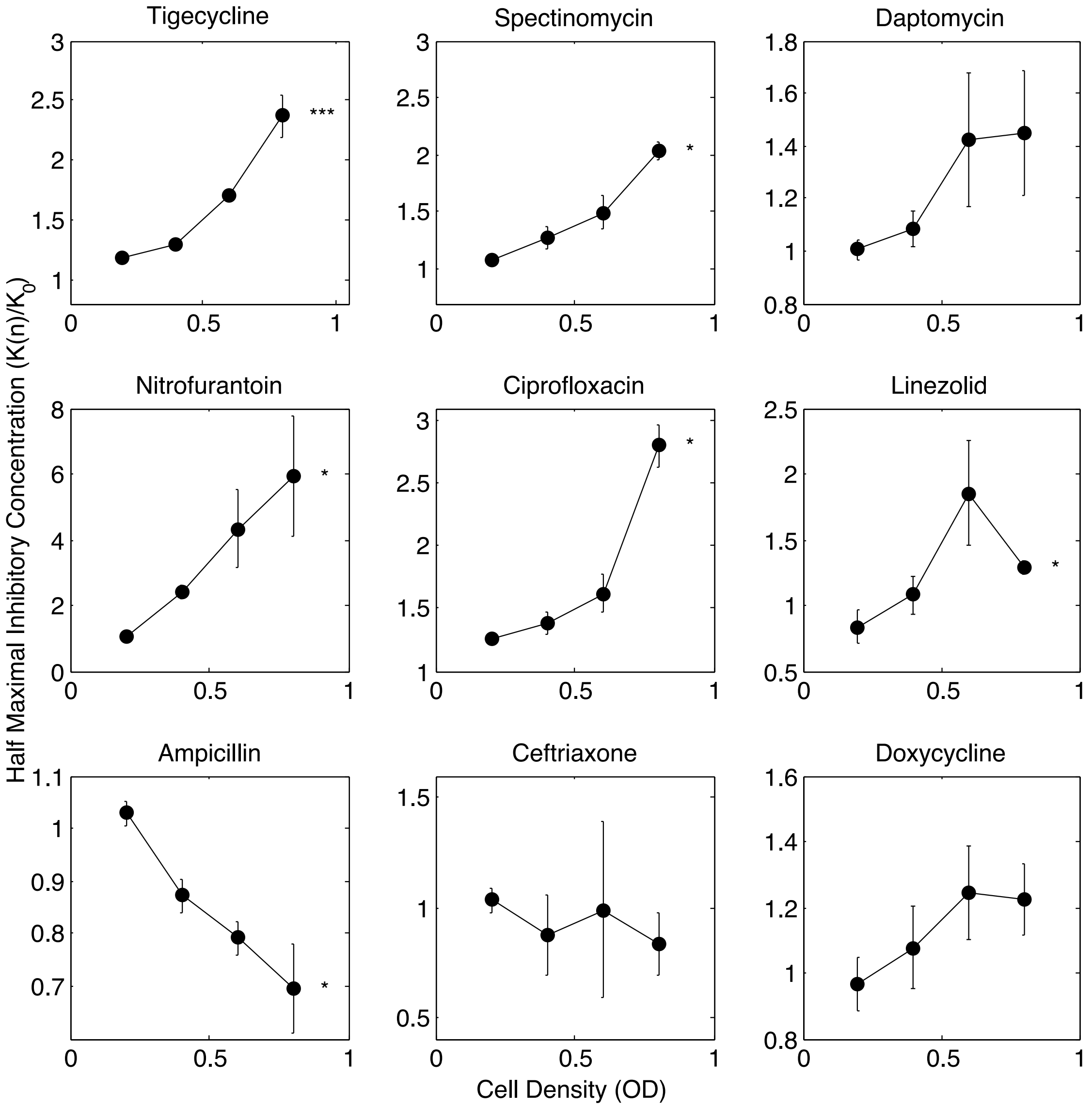

Figure C in S1 Text: Cell density modulates the half maximal inhibitory concentration (K) for multiple drugs. Half maximal inhibitory concentration (relative to its low density value, K_0) for the same drugs as in Figure 2. Note that the vertical scales are not the same for all figures. From left to right: Tigecycline, Spectinomycin, Daptomycin (top row); Nitrofurantoin, Ciprofloxacin, Linezolid (middle row); Ampicillin, Ceftriaxone, Doxycycline (bottom row). Statistically significant differences between growth at lowest and highest densities (0.2 and 0.8), intermediate densities (0.4 and 0.6), or both are indicated by *, **, and ***, respectively. Error bars are +/- standard errors of the fitted K parameter at each density.

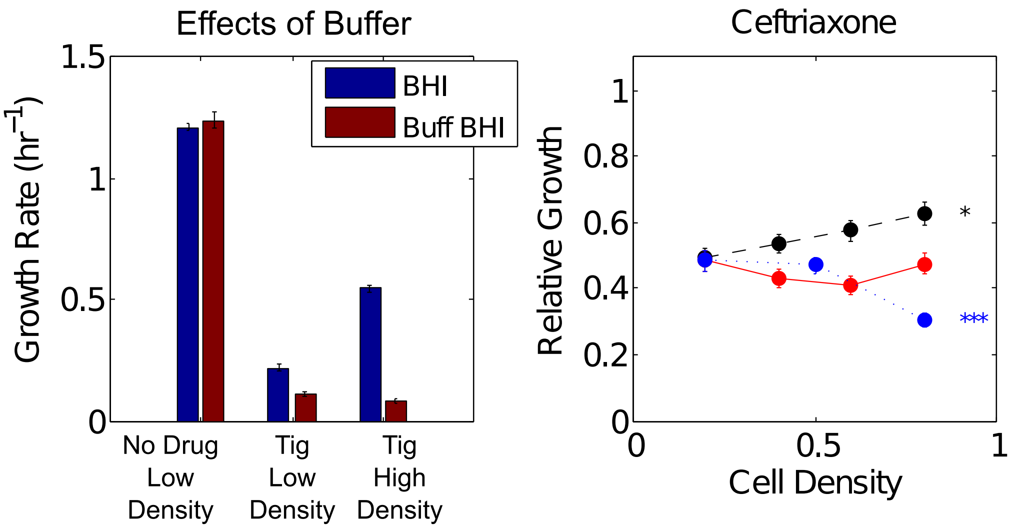

Figure D in S1 Text: Buffered media does not affect drug-free growth but alters density dependence of ceftriaxone. A. Left: Growth rate of low density cultures in the absence of drug in regular media (blue) and highly buffered media (red). Growth in the presence of tigecycline at low densities (middle) and high densities (right). Growth in buffered and regular media did not differ significantly in the absence of drug, but in the presence of tigecycline, buffered media leads to slightly (low density) and then dramatically (high density) decreased growth rate. **B.** Relative growth rate of cells exposed to ceftriaxone (50 ug/mL) in regular media (red), strongly buffered media (black, dashed), and in low-density (OD=0.2) cultures with externally modulated pH (blue, dotted). In the latter case, buffer was supplemented with HCl to achieve pH = 7.5, 6.8, and 6.0, which correspond to pH of steady state cultures held at OD=0.2, 0.5, and 0.8, respectively. See also Figure 3.

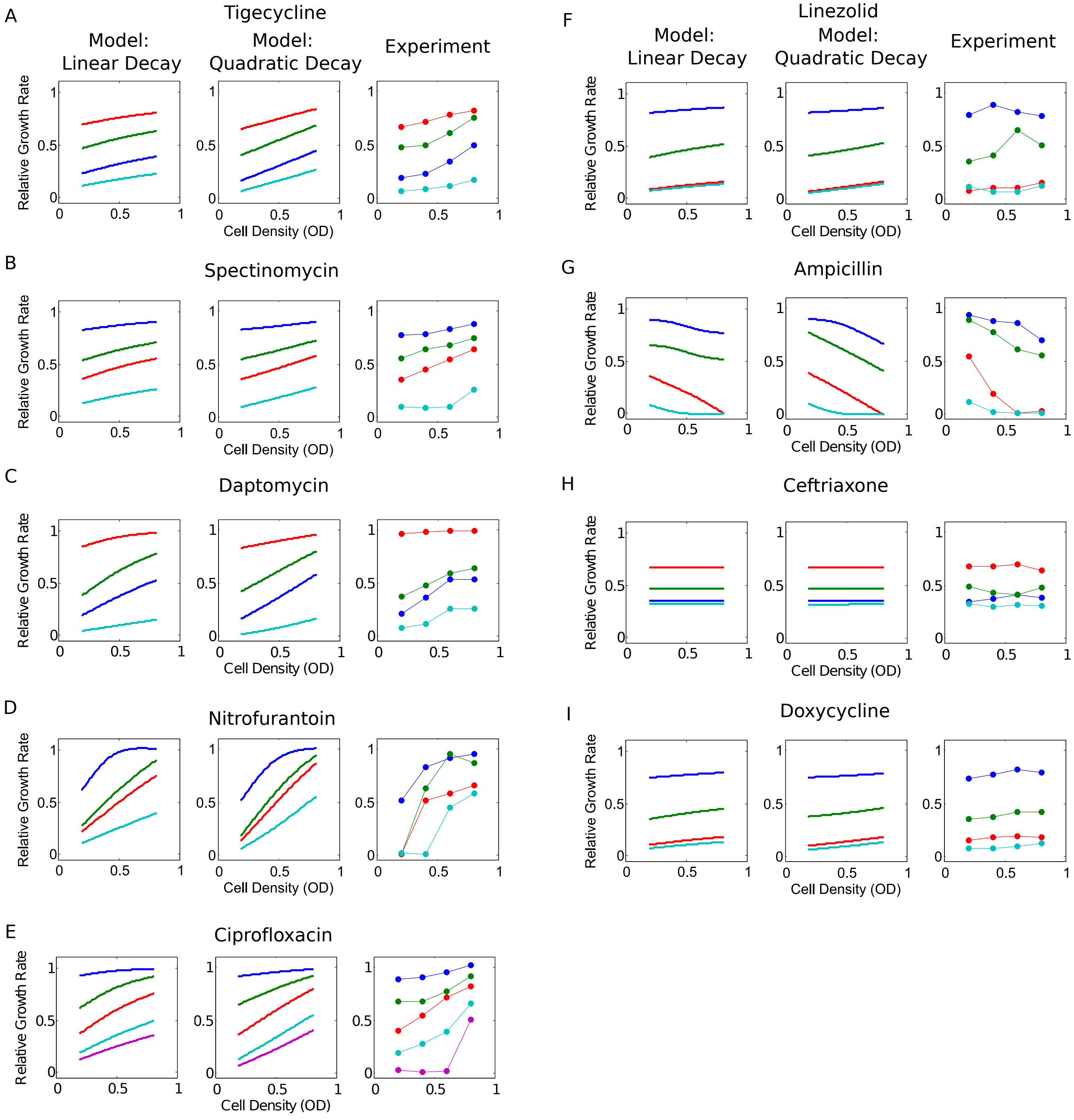

Figure E in S1 Text: Linear and quadratic decay models capture qualitative features of density-dependent growth. For each drug, the panels compare the linear decay model (left), the quadratic decay model (middle), and the experimental data (right) for relative per capita growth rate as a function of cell density. Different colors represent different drug concentrations (same is in Figure 2, main text). For tigecycline, the parameter ε was fit from turbidostat data, while K_0 (IC_{50}) and h were determined independently from low density dose response curves (see Figure F in S1 Text). For the remaining drugs, ε, K_0, and h were fit simultaneously from the turbidostat data because low density dose response curves were not measured, though similar results were obtained if K_0 and h were determined from the OD=0.2 data alone.

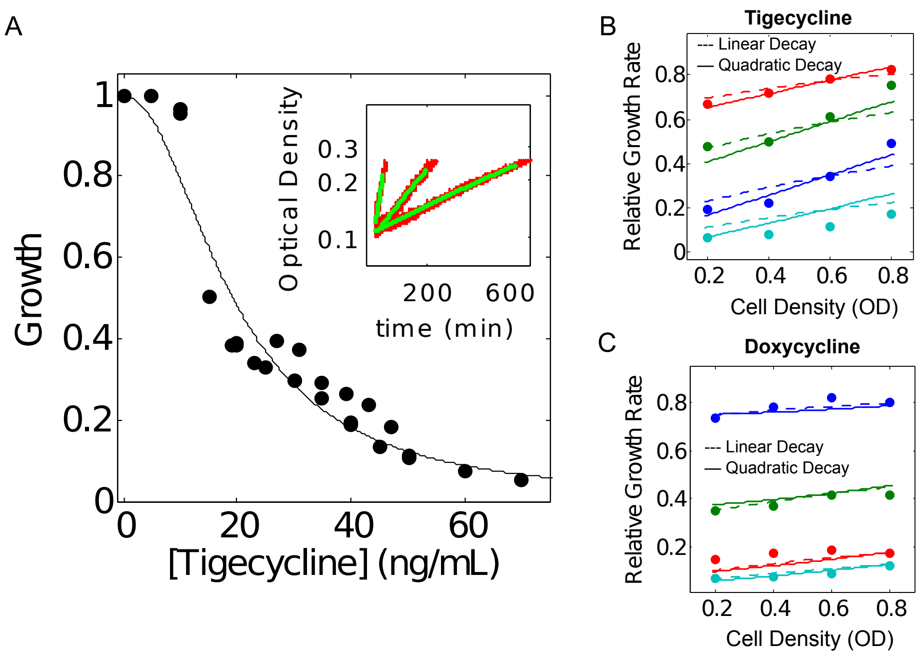

Figure F in S1 Text: Model fits to experimental data. A. Relative growth rate of V583 populations as a function of tigecycline in regular media (BHI). Circles, growth rates measured in low-density (OD<0.2) exponentially growing populations. Solid line, fit to dose response curve $g = (1 + (D/K)^h)^{-1}$. For tigecycline in regular media, K =19.3±1 and Hill coefficient h=2.1±0.1. We also found a similar dose response curve for tigecycline in highly buffered media, but K is slightly lower (K=11.1±0.4 and Hill coefficient h=2.1±0.2). For each concentration, we estimated growth rate by fitting the time series of optical density in the low density region to an exponential function using nonlinear least squares fitting. Inset: example low-density growth curves for [Tigecycline]=10, 40, and 60 ng/mL. Red points: OD measurements. Green lines: fit to exponential function. **B. and C**. Comparison of linear (dashed lines) and quadratic (solid lines) decay decay models to experimental data (circles) for tigecycline (B) and doxycycline (C). The former is best fit by the quadratic model, while the latter is best fit by the linear model according to AIC model selection (see Table B of S1 Text).

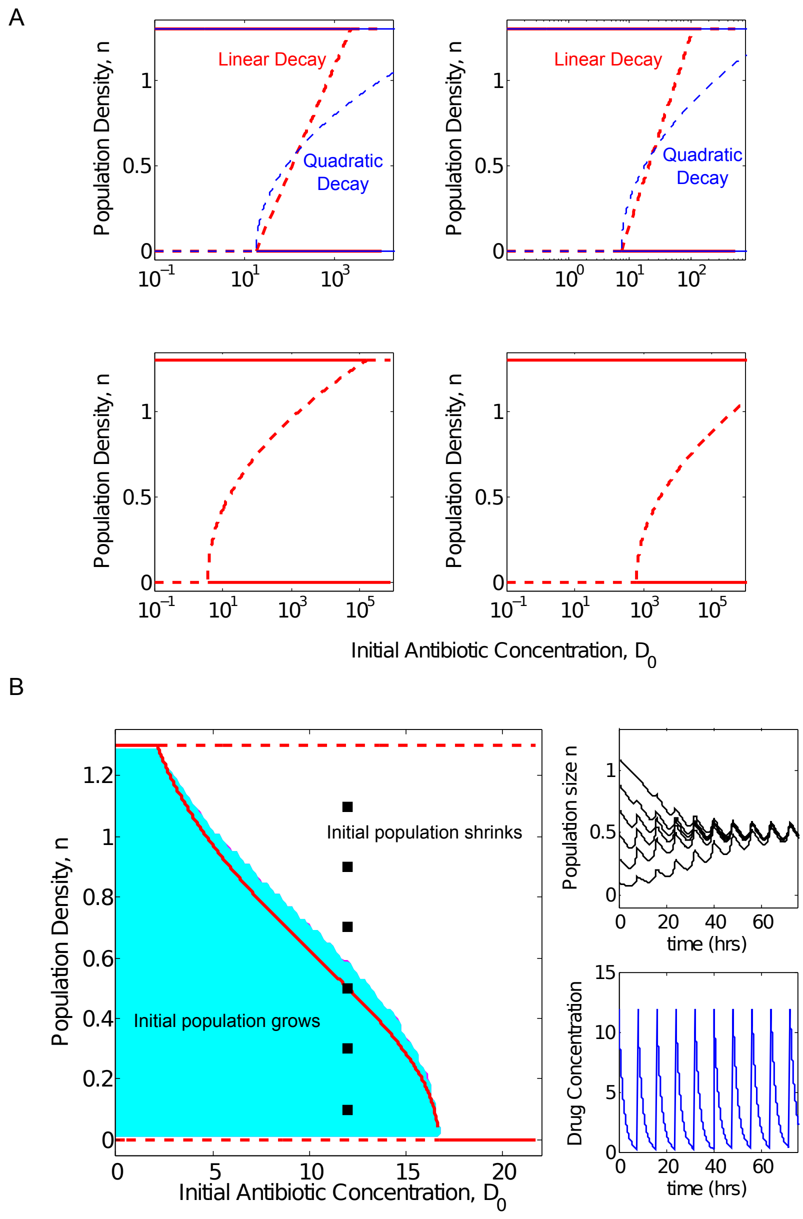

Figure G in S1 Text: PK/PD Phase Diagrams. A. Top panels show phase diagrams (i.e. bifurcation plots) for linear decay model (red) and quadratic decay model (blue) based on tigecycline growth data for low (left) and high (right) maximum kill rate. Left panel: g_{min}=-0.05; right panel, g_{min}=-1. Solid lines, stable fixed points of population density (theory). Dashed lines, unstable fixed points (theory). The curved dashed line is the phase boundary (separatrix) indicating the critical density above which a population will survive. The decay rate is k_d = ½ in both plots. Lower panels show phase diagrams (quadratic model) for low (k_d = ¼, left) and high (k_d = 1, right) values of the native drug decay rate. In both plots, g_{min}=-0.05. ε=0.9 (quadratic model) or ε=0.5 (linear model) and h=2 in all plots. **B.** Main panel: Theoretical (solid and dashed lines) and numerical (shaded region) phase diagrams indicate treatment outcomes in PK/PD model as a function of initial cell density (ranging from 0 to the carrying capacity, C=1.3) and initial antibiotic concentration D_0. Solid red lines, stable fixed points of population density (theory). Dashed red lines, unstable fixed points (theory). Shaded regions indicate that initial population grows, while unshaded regions indicate that initial population shrinks in numerical simulations of the PK/PD model. Upper right inset: numerical solution of PK/PD equations for five different initial densities (indicated by black squares on the phase diagram). Lower inset: temporal dynamics of antibiotic concentration. For numerical phase diagram and simulations, g_{min}=-0.1 (units of hrs^{-1}) and ε=-0.2.
